# Effect of Neoadjuvant Hormonal Therapy on the Postoperative Course for Patients Undergoing Robot-Assisted Radical Prostatectomy

**DOI:** 10.3390/jcm12093053

**Published:** 2023-04-22

**Authors:** Mahmoud Farzat, Peter Weib, Iurii Sukhanov, Josef Rosenbauer, Christian Tanislav, Florian M. Wagenlehner

**Affiliations:** 1 Department of Urology and Robotic Urology, Diakonie Klinikum Siegen, Academic Teaching Hospital of the University of Bonn, 57074 Siegen, Germany; 2 Department of Urology, Pediatric Urology and Andrology, Justus-Liebig University of Giessen, 35392 Giessen, Germany; 3 Department of Geriatric and Neurology, Diakonie Klinkum Siegen, Academic Teaching Hospital of the University of Bonn, 57074 Siegen, Germany

**Keywords:** prostate cancer, RARP, neoadjuvant hormonal therapy

## Abstract

Objectives: Neoadjuvant hormonal therapy (NHT) preceding robot-assisted radical prostatectomy (RARP) may be beneficial in high-risk cases to facilitate surgical resection. Yet, its improvement in local tumor control is not obvious. Its benefit regarding overall cancer survival is also not evident, and it may worsen sexual and hormonal functions. This study explores the effect of NHT on the perioperative course after RARP. Methods: In this study, 500 patients from a tertiary referral center who underwent RARP by a specialized surgeon were retrospectively included. Patients were divided into two groups: the NHT (*n* = 55, 11%) group, which included patients who received NHT (median: 1 month prior to RARP), and the standard non-NHT (NNHT) group (*n* = 445, 89%). Demographic and perioperative data were analyzed. Postoperative results, complications, and readmission rates were compared between the groups. Results: NHT patients were heterogeneous from the rest regarding cancer parameters such as PSA (25 vs. 7.8 ng/mL) and tumor risk stratification, and they were more comorbid (*p* = 0.006 for the ASA score). They also received fewer nerve-sparing procedures (14.5% vs. 80.4%), while the operation time was similar. Positive surgical margins (PSM) (21.8% vs. 5.4%) and positive lymph nodes (PLN) (56.4% vs. 12.7%) were significantly higher in the NHT group compared to the non-NHT (NNHT) group. Hospital stay was equal, whereas catheter days were 3 days longer in the NHT group. NHT patients also suffered more minor vesicourethral-anastomosis-related complications. Major complications (*p* = 0.825) and readmissions (*p* = 0.070) did not differ between groups. Conclusion: Patients receiving NHT before RARP did not experience more major complications or readmissions within 90 days after surgery. Patients with unfavorable, high-risk tumors may benefit from NHT since it facilitates surgical resection. Randomized controlled trials are necessary to measure the advantages and disadvantages of NHT.

## 1. Introduction

Neoadjuvant hormonal therapy may be beneficial to facilitate surgical resection in locally advanced prostate cancers prior to robot-assisted radical prostatectomy (RARP). It may also statistically improve pathologic findings [1]. NHT has shown efficacy in tumor downsizing [2], yet this does not often lead to downstaging [3]. It is well known that NHT does not improve overall survival, yet it helps with local tumor control [3]. Radiotherapists studied the effects of 3 and 6 months of NHT combined with radiotherapy on locally advanced prostate cancer [4]. Its use has also been studied in combination with cryotherapy [5]. Some authors found NHT to facilitate laparoscopic radical prostatectomy (LRP) and RARP, reduce the positive surgical margin rate, and accelerate the recovery of short-term urinary control [6]. A prolonged NHT of 8 months was found to have no additional benefits compared to the 3-month period of NHT in a laparoscopic radical prostatectomy cohort, and both regimes were found to lower positive surgical margin rates compared to patients who underwent surgery without previous therapy [7]. Furthermore, the sexual function two years after RARP in patients receiving NHT may not be worse than that of patients receiving RARP without NHT [8]. Moreover, hormonal therapy is associated with higher comorbidity, especially in patients with risk factors [9,10]. Regarding perioperative morbidity, NHT patients are found to be equivalent to non-NHT patients, with lower positive surgical margins and PSA recurrence rates [11]. In another study, it was found that NHT reduces prostate-cancer-related deaths, although the authors concluded that this effect may be explained by the early addition of radiotherapy post-surgery [12]. Patients receiving combined neoadjuvant hormonal chemotherapy experienced a higher rate of serious complications: 13.3% [13]. NHT alone deteriorates sexual and hormonal function after RARP [14]. All of this results in NHT not being recommended in urological guidelines [15]. Still, urologists prescribe NHT to the highest-risk patients prior to prostatectomies to facilitate the procedure, shorten OR times, reduce blood loss, and reduce positive surgical margins [16]. Conversely, some authors found that NHT increases operating times without further comorbidity [17]. This study investigates the impact of NHT on the perioperative course after RARP in a large cohort of 500 consecutive cases without any exclusion criteria performed by a single surgeon in a tertiary hospital.

## 2. Material and Methods

### 2.1. Surgical Procedure and Setting

All procedures (*n* = 500) were completed transperitoneally with Da Vinci X^®^ Surgical Systems (Intuitive Surgical, Sunnyvale, CA, USA). Patients were positioned in 25° Trendelenburg. A capnoperitoneum of 5–8 mmHg was used routinely. In very obese patients, a capnoperitoneum of 12 mmHg was necessary to complete the procedure. Pelvic lymphadenectomy was performed in all cases. It included the external iliac, obturator, and epigastric lymph nodes. No intra-abdominal drainage was inserted. Prior to skin incisions, intravenous single-shot antibiotics were administered. In patients with pre-existing urinary tract infections or catheter-associated infections, urine cultures were obtained preoperatively. Accordingly, perioperative antibiotics were administered for a period of 5 days, starting one day before surgery. The vesicourethral anastomosis (VUA) was performed in a one-layer fashion with a continuous circumferential double-armed barbed suture. A ventral reconstruction was utilized rarely. Dorsal reconstruction was completed via a one-layer Rocco stitch. After completion, patients received anastomosis water-tightness tests with 200–300 mL of sterile NaCl intra-operatively. All patients received transurethral (TUC) and suprapubic (SPC) catheters. The transurethral catheter was removed on the first postoperative day (POD1). On POD3, patients were allowed to urinate naturally. The suprapubic catheter was removed after one day when micturition was successful without post-void residual urine. In cases of primary extravasation on cystography, patients were discharged with catheters, and the catheters were later removed during an outpatient visit.

### 2.2. Participants and Methods

A total of 500 consecutive patients from a prospectively collected database who underwent RARP between 04/2019 and 08/2022 performed by a specialized surgeon due to locally confined (pT2; *n* = 295; 59.4%) and locally advanced prostate cancer (pT3-4; *n* = 203; 40.6%) were included in the analysis.

A total of 55 patients with high-risk tumor characteristics were given LHRH agonists for a period of 1–3 months prior to being referred to surgery (median: 1 month). We ran propensity score matching to match the 55 NHT cases. The use of oncological parameters such as the Gleason score or prostate-specific antigen (PSA) was not feasible since NHT patients were notably heterogeneous from the rest of the 455 cases within our cohort. Here, we used a ratio of 0.6 for propensity score matching. Likewise, the use of demographic parameters such as age, the American Association of Anesthesiology morbidity score (ASA), prostate volume in the transrectal ultrasound (TRUS), body mass index (BMI), preoperative hemoglobin (Hgb), the International Prostate Symptom Score (IPSS), and the International Index of Erectile Function (IIEF) questionnaire also resulted in no suitable matching cases when using a propensity score matching ratio of 0.6. Hence, we compared *n* = 55 NHT (NHT group) patients with *n* = 455 patients not receiving neoadjuvant hormonal therapy (NNHT group) to mirror real-life scenarios. Demographic, intraoperative, and postoperative data were analyzed and compared between groups. All the aforementioned variables were included. Postoperative complications were graded using the Clavien–Dindo classification [18]. Complications and the readmission rate were noted for the first 90 days postoperatively.

This study’s design comprises a retrospective cohort study. Statistical analysis was performed using SPSS^®^ v27. Categorical variables were summarized as frequencies (percentage) and continuous variables as mean ± standard deviation and median values. The Kolmogorov–Smirnov one-sample test was used to verify normal distribution. Matched-pair analysis using the independent T-test for parametric numeric variables and the Mann–Whitney U test for nonparametric variables was performed. Pearson’s chi-square test was also used to compare relative frequencies.

### 2.3. Ethics Statement

The study was conducted in accordance with the ethical standards of the Declaration of Helsinki and approved by the ethics committees of the medical association Westfalen-Lippe and Wilhelm’s University of Münster (2022-585-f-S).

## 3. Results

Baseline Parameters: NHT patients were slightly more overweight and clearly more morbid than NNHT patients (BMI *p* = 0.045 and ASA *p* = 0.006). They had significantly larger prostates in TRUS (medians: 50 mL versus 43 mL), but the IPSS scores were not significantly different (prostate volume *p* < 0.001 and IPSS *p* = 0.057). Even though, statistically, the Gleason score distribution was comparable between groups (*p* = 0.912), oncological parameters were clearly in favor of the NNHT group. The median PSA was 25 ng/mL in NHT versus 7.8 ng/mL in NNHT. While more than three-quarters (80.4%) of NNHT group patients were operated upon using the nerve-sparing technique, the majority of NHT patients were not (*n* = 47; 85%) (*p* < 0.001) (Table 1).

Intraoperative data: The median console operating time was 10 min longer in the NHT group (150 min versus 140 min). Nevertheless, this did not result in statistical differences (*p* = 0.519). In *n* = 43/55 of the NHT patients, the pathological report did not report a Gleason score but rather reported regression grades after Helpap due to changes seen in the prostate tissue via hormonal therapy. The difference in the postoperative Gleason score distribution is not applicable. Nonetheless, tumors in the NHT patients were, as expected, locally advanced in 81.2% of cases compared to 35.4% in the standard group (*p* = 00.2). Consequently, positive surgical margins were significantly higher in the NHT group, with 21.8% versus 5.4% in the standard group (*p* < 0.001). Furthermore, more than half of the NHT patients had lymphogenic metastases (56.4%) compared to 12.6% in the standard group. Although all patients had the same length of hospital stay (median: 5 days), the NHT patients had their suprapubic catheters for 3 days longer (7 versus 4 days; *p* = 0.013). Further details are shown in Table 2.

Complications and Readmissions: NHT patients experienced more minor complications than NNHT patients (29% versus 13%; *p* = 0.002). The most common adverse event was acute urinary retention (AUR) (*n* = 5, 9%), followed by secondary vesicourethral anastomosis leakage (VUAL) (*n* = 4, 7.2%) details in Table 3. In those cases, the initial micturition‘s trial was uneventful, and those patients were able to empty their bladders with sufficient flow and without relevant residual urine. However, two to three weeks after discharge, they presented with micturition-associated abdominal pain again. Via ultrasound, free intra-abdominal fluid was detected; on cystography, a leakage in the vesicourethral anastomosis was identified. Therefore, a transurethral catheter was inserted, and antibiotics were administered. In all cases, the symptoms were resolved with conservative treatment, and the catheters were removed when subsequent cystography proved the integrity of the vesicourethral anastomosis. Nonetheless, statistical analysis showed no difference in major complications and readmissions (*p* = 0.825). Two revisions were carried out in NHT patients. The first revision was carried out due to intestinal obstruction caused by a large bilateral inguinal hernia, despite being repaired during RARP using a mesh. In all patients with simultaneous inguinal hernias, meshes with a minimum of 3 cm exceeding the abdominal wall defect were used to repair the hernias. Thereafter, meshes were fixed with Monocryl 3° separate sutures. In this particular case, the mesh was found in the scrotum and was removed intraoperatively. The second revised patient suffered from intestinal obstruction symptoms on the third day postoperatively. Physical examination revealed an incisional hernia in the median mini-laparotomy. A revision was carried out in which the fascia’s defect was repaired, and the patient could be discharged after two days. Notably, neither symptomatic lymphocele nor thromboembolic accidents were recorded in the NHT patients. All grade IIIa complications were seen in the NNHT group. Overall, 28 patients had to be readmitted after discharge within 90 days after RARP. Despite a trend toward a higher incidence in the NHT group (*n* = 6/55, 10.9%, vs. *n* = 22/445, 4.9%, in the NNHT group), statistical analysis showed no statistical difference between the two study groups (*p* = 0.071), further datails in Table 3. 

## 4. Discussion

NHT prior to prostatectomy or radiotherapy provides substantial benefits regarding local tumor control and may have a positive impact on survival [3]. However, hormonal therapy is associated with significant side effects, such as hot flashes, gynecomastia, and cardiovascular incidents. The main finding of our study is that the NHT patients did not experience major complications or readmissions after RARP. Our findings are in line with Naiki et al., who found in their laparoscopic prostatectomy series that the perioperative morbidity of NHT patients was equivalent to that of non-NHT patients [6]. On the contrary, radiotherapists reported hormonal therapy to be associated with higher comorbidities, especially in patients with cardiovascular risk factors [4,5]. Notably, patients treated with radiotherapy usually receive hormonal therapy for longer periods (6, 18, or 36 months). Many authors have reported the oncological effect of NHT on cancer-specific survival and biochemical-free survival [3,7], yet this is the first study, with this number of patients without any exclusion criteria, primarily investigating the impact of short-term neoadjuvant hormonal therapy on the perioperative course after RARP.

Another major finding of our study is that NHT patients suffered from more minor complications after RARP, such as AUR or secondary VUAL, due to prolonged convalescence postoperatively. However, those patients might have been inoperable due to a largely fixed prostate or an extended extracapsular tumor infiltration. They also may have experienced many more complications while undergoing prostate removal without hormonal therapy or while undergoing prolonged hormonal therapy alone. Androgen deprivation therapy is combined with significant clinical benefits in patients with locally advanced prostate cancer. Such local tumor control may improve a patient’s quality of life. Nevertheless, its impact presenting a significant survival advantage is still debatable [3]. A trend toward higher vesicourethral anastomosis leakage in NHT patients (7.2% vs. 1.5%) can be suggested. Statistical analysis was not possible due to the small sample size. First, this might be explained by extended bladder neck resection and more challenging vesicourethral anastomosis in NHT patients with more aggressive tumors. Secondly, it can be explained by the reduced quality of urethral and bladder neck tissues involved in the anastomosis, with possible alterations due to tumor infiltration and changes caused by hormonal therapy. Both reasons may have prolonged the healing of the anastomosis and resulted in higher VUAL rates.

Moreover, NHT patients, despite being more ill than their counterparts, had a similar median length of hospital stay compared to that of the NNHT patients (median: 5 days). The NHT patients did not show major complications or higher rates of readmissions and had a similar median length of hospital stay compared to that of the NNHT patients. On the other hand, the median of urinary catheter days among NHT patients was 7 versus 4 days in the standard group. This is explained by the already short catheter days within our cohort (median: 4 days); additionally, in such patients with locally advanced tumors, the bladder neck could not be spared, leading to the catheters being left in place for a longer time, as shown in the other study [19]. Authors used to report the results of NHT studies categorized according to risk group. The NHT patients in our study were completely heterogeneous from the rest. This was preoperatively obvious since 78% of NHT patients were in the D’Amico high-risk group compared to 24.9% of NNHT men. Due to the highly individual states, finding equal matches was not applicable within our cohort.

In our study, the overall positive surgical margins were low at 7.2%. Nevertheless, they were higher in NHT patients (21.8% compared to 5.4% in the NNHT group). This is in contrast to others who noted lower positive surgical margins and a lower PSA recurrence rate in NHT patients [6]. This might be somewhat explained by the trial of nerve-sparing in sexually motivated men harvesting aggressive tumors. Moreover, some of those tumors were, despite hormonal therapy, surgically incurable. Additionally, the men in our study received hormonal therapy for significantly shorter periods than in other reports [3]. The operating time in our study was the same for both groups, suggesting that NHT did not prolong the procedure, in contrast to other findings [12]. This might be due to the fact that NHT reduces both prostate size and infiltrations of the bladder neck.

Furthermore, the median BMI of NHT cases was significantly higher than that of standard NNHT cases (30 vs. 28, respectively) (*p* = 0.045). However, obesity in patients undergoing RARP is associated with more difficult intraoperative courses, elevated rates of case abortion, unfavorable postoperative outcomes, increased operating time, reduced nerve-sparing technique, and higher rates of positive surgical margins [20,21,22,23]. Within our cohort, the operating time and catheter days were longer in NHT patients, which may also be influenced by higher BMIs and not merely the high-risk tumor characteristics or the use of neoadjuvant hormonal therapy.

The strength of our investigation is the large number of patients included and the detailed analysis of their pre- and postoperative parameters and outcomes. However, limitations must be taken into account. The main limitation of our study is its retrospective design. Secondly, we did not report long-term biochemical-free survival due to a lack of long-term follow-up data caused by the regulations of the national health care system, in which follow-up is not conducted by tertiary referral centers. However, the biggest limitation is that we did not compare patients with the highest-risk tumors, such as those who received previous hormonal therapy, to find out what could have happened. Furthermore, NHT is known to cause pseudo-negative surgical margins and pseudo tumor-free lymoh nods. Furthermore, it will impede accurate pathological reporting due to the hormonal modification of prostatectomy specimens after RARP.

## 5. Conclusion

NHT does not put patients at an elevated risk for increased complications or readmissions after RARP. Patients with unfavorable, high-risk tumors may benefit from NHT since it might improve local tumor control and facilitate surgical resection. Further randomized controlled trials are indispensable to measure the advantages and disadvantages of NHT prior to RARP.

## Figures and Tables

**Table 1:** Analysis of demographic, clinical, and preoperative characteristics between groups.

	Total Cohort (500)	No previous Hormonal Therapy N = 445	Neoadjuvant Hormonal Therapy N = 55	*p*-Value
Age (year) Mean ± SD Median	66.8 ± 7.1 68	66.6 ± 7.2 68	68.1 ± 6.1 70	0.335
BMI (kg/m^2^) Mean ± SD Median	28.4 ± 4.3 28	28 ± 4.3 28	30.5 ± 5.1 30	0.045
ASA-score 1 2 3	99 (19.8) 317 (63.4) 84 (16.8)	91 (20.4) 288 (64.7) 66 (14.8)	8 (14.5) 29 (52.7) 18 (32.7)	0.006
Preoperative Hgb (g/dL) Mean ± SD median	14.7 ± 1.18 14.8	14.7 ± 1.3 14.9	14.1 ± 1.27 14.1	0.603
IPSS Mean ±SD median	11.4 ± 8.3 8.3	10.7 ± 7.9 9.5	16.7 ± 9.8 16.5	0.057
IIEF	15.2 ± 8.7 17	16 ± 8.4 17	9.9 ± 8 6	0.183
Mean (SD)				
median				
Initial PSA (ng/mL) Mean ± SD median	14.8 ± 24.5 8	11.1 ± 14.2 7.8	45 ± 50 25	<0.001
Prostate-Volume (mL) Mean ± SD median	49 ± 28 43	48 ± 22 43	59 ± 46 50.5	<0.001
D’Amico Risk Classification Low risk Intermediate risk High risk	117 (23.4) 229 (45.8) 154 (30.8)	114 (25.6) 220 (49.4) 111 (24.9)	3 (5.5) 9 (16.4) 43 (78.2)	<0.001
Preoperative Gleason score 5 6 3 + 4 4 + 3 8 9 10 Unclassified *	1 (0.2) 140 (28) 176 (35.2) 59 (11.8) 82 (16.4) 36 (7.2) 5 (1.0) 1 (0.2)	0 136 (30.6) 169 (38) 52 (11.7) 66 (14.8) 20 (4.5) 2 (0.4) 0	1 (1.8) 4 (7.3) 7 (12.7) 7 (12.7) 16 (29.1) 16 (29.1) 3 (5.5) 1 (1.8)	0.912
previous surgical treatment (TUR-P)	34 (6.8)	27 (6)	7 (12.7)	0.067
Nerve Sparing Bilateral Unilateral No	374 (69.4) 19 (3.8) 134 (26.8)	340 (76.4) 18 (4) 87 (19.6)	7 (12.7) 1 (1.8) 47 (85.5)	<0.001

Categorical data are presented as numbers and %; SD: standard deviation; *: patient received hormonal therapy prior to prostate biopsy, BMI: body mass index; ASA: American Association of Anesthesiology comorbidity score; Hgb: hemoglobin; IPSS: International Prostate Symptom Score; IIEF: International Index of Erectile Function; PSA: prostate-specific antigen; TUR-P: transurethral resection of the prostate.

**Table 2:** Intra- and postoperative data and pathological findings for all groups.

	Total (500)	No Previous Hormonal Therapy N = 445, 89%	Neoadjuvant Hormonal Therapy N = 55, 11%	*p*-Value
OR-Time Mean ± SD median	151 ± 45 140	150 ± 45 140	155 ± 48 150	0.519
Prostate weight (g) Mean ± SD median	61± 25.6 55	60 ± 24 54	71± 30 66	0.038
Pathological stage 0 pT1 pT2 pT3 pT4	1 (0.2) 1 (0.2) 295 (59) 183 (36.6) 20 (4.0)	0 1 (0.2) 286 (64.2) 149 (33.4) 9 (2)	1 (1.8) 0 9 (16.3) 34 (61.8) 11 (20)	0.002
Postoperative Gleason score 6 3 + 4 4 + 3 8 9 10 Unclassified *	28 (5.6) 282 (56.4) 89 (17.8) 26 (5.2) 29 (5.8) 1 (0.2) 45 (9.0)	28 (6.3) 280 (62.9) 83 (18.7) 26 (5.8) 25 (5.6) 1 (0.2) 2 (0.4)	0 2 (3.6) 6 (10.9) 0 4 (7.3) 0 43 (78.2)	<0.001
Positive surgical margins	36 (7.2)	24 (5.4)	12 (21.8)	<0.001
Number of Lymph nodes Mean ± SD median	19.6 ± 7.4 18	19.6 ± 7.4 18	20.1 ± 7.8 19	0.802
Positive Lymph nodes	87 (17.4)	56 (12.6)	31 (56.4)	<0.001
Hgb-Difference (g/dL) Mean ± SD median	2.5 ± 4.8 2.6	2.7 ± 1.3 2.7	2.64 ± 1.3 2.4	0.817
Transfusion	7 (1.2)	6 (1.3)	1 (1.8)	0.785
hospitalization (days) Mean ± SD median	5.6 ± 1.5 5	5.57 ± 1.5 5	5.98 ± 1.5 5	<0.100
Catheter days Mean ± SD median	6.9 ± 4.7 5	6.7 ± 4.5 4	8.8 ± 5.6 7	0.013
Catheter removed before discharge	368 (73.6)	333 (74.8)	35 (63.6)	0.076

Categorical data are presented as numbers%; SD: standard deviation; Hgb: hemoglobin. *: in patients received systematic therapies like neoadjuvant hormonal therapy Gleason Score is not applicable.

**Table 3:** 90-days complications and readmissions.

Complications in Detail			Total (*n* = 500)	No Previous Hormonal Therapy N = 445, 89%	Neoadjuvant Hormonal Therapy N = 55, 11%	*p*-Value
	Minor		74 (14.8)	58 (13)	16 (29.1)	0.002
Minor	CDI 51 (10.2)	VTE	4 (0.8)	4 (0.9)	0	
		Elevated Labor Parameter	6 (1.2)	2 (0.4)	4 (7.2)	
		AUR	28 (5.6)	23 (5.1)	5 (9)	
		Diverse	13 (2.6)	12 (2.6)	1 (1.8)	
	CD II 23 (4.6)	Secondary VUAL *	11 (2.2)	7 (1.5)	4 (7.2)	
		UTI	11 (2.2)	9 (2)	2 (3.6)	
		Hematoma requiring Transfusion	1 (0.2)	1 (0.2)	0	
	Major		21 (4.2)	19 (4.2)	2 (3.6)	0.825
Major	CD III a 12 (2.4)	NSTEMI	1 (0.2)	1 (0.2)	0	
		Hiatus Hernia	1 (0.2)	1 (0.2)	0	
		Symptomatic Lymphocele	10 (2.0)	10 (2.2)	0	
	CD III b 8 (1.6)	Revision	5 (1.0)	3 (0.6)	2 (3.6)	
		UUTO	3 (0.6)	3 (0.6)	0	
	CD VI 1 (0.2)	Rhabdomyolysis	1 (0.2)	1 (0.2)	0	
		Readmissions *	28 (5.6)	22 (4.9)	6 (10.9)	0.070

* Some patients presented to the emergency unit had mixed AUR + VUAL + UTIs; we listed the most serious complaint. Categorical data are presented as numbers%; VTE: venous Thromboembolism; AUR: acute urinary retention; VUAL: vesicourethral anastomosis leakage; UTI: urinary tract infection; NSTEMI: non-ST elevating myocardial infarct; UUTO: upper urinary tract obstruction.

## Data Availability

Data supporting results is available when requested.

## List of Abbreviations

ADT	Androgen deprivation therapy
ASA	American Association of Anesthesiology comorbidity score
AUR	Acute urinary retention
BMI	Body mass index
CD	Clavien–Dindo classification of postoperative complication
HBG	Hemoglobin
IIEF	International Index of Erectile Function
IPSS	International Prostate Symptom Score
NHT	Neoadjuvant hormonal therapy
NSTEMI	Non-ST-elevation myocardial infarction
POD	Postoperative day
PSA	Prostate-specific antigen
PSM	Positive surgical margins
TUR-P	Transurethral resection of the prostate
RARP	Robot-assisted radical prostatectomy
SPC	Suprapubic catheter
TUC	Transurethral catheter
LOS	Length of hospital stay
LRP	Laparoscopic radical prostatectomy
UTI	Urinary tract infection
VTE	Venous thromboembolism
UUTO	Upper urinary tract obstruction
VUA	Vesicourethral anastomosis
VUAL	Vesicourethral anastomosis leakage
SVUAL	Secondary vesicourethral anastomosis leakage
